# Arterial Dysfunction but Maintained Systemic Blood Pressure in Cavin-1-Deficient Mice

**DOI:** 10.1371/journal.pone.0092428

**Published:** 2014-03-21

**Authors:** Karl Swärd, Sebastian Albinsson, Catarina Rippe

**Affiliations:** Department of Experimental Medical Science, Lund University, Lund, Sweden; University of Southampton, United Kingdom

## Abstract

Caveolae are omega-shaped plasma membrane micro-domains that are abundant in cells of the vascular system. Formation of caveolae depends on caveolin-1 and cavin-1 and lack of either protein leads to loss of caveolae. Mice with caveolin-1 deficiency have dysfunctional blood vessels, but whether absence of cavin-1 similarly leads to vascular dysfunction is not known. Here we addressed this hypothesis using small mesenteric arteries from cavin-1-deficient mice. Cavin-1-reporter staining was intense in mesenteric arteries, brain arterioles and elsewhere in the vascular system, with positive staining of both endothelial and smooth muscle cells. Arterial expression of cavin-1, -2 and -3 was reduced in knockout (KO) arteries as was expression of caveolin-1, -2 and -3. Caveolae were absent in the endothelial and smooth muscle layers of small mesenteric arteries as determined by electron microscopy. Arginase, a negative regulator of nitric oxide production, was elevated in cavin-1 deficient arteries as was contraction in response to the α_1_-adrenergic agonist cirazoline. Detailed assessment of vascular dimensions revealed increased media thickness and reduced distensibility, arguing that enhanced contraction was due to increased muscle mass. Contrasting with increased α_1_-adrenergic contraction, myogenic tone was essentially absent and this appeared to be due in part to increased nitric oxide production. Vasomotion was less frequent in the knock-out vessels. In keeping with the opposing influences on arterial resistance of increased agonist-induced contractility and reduced myogenic tone, arterial blood pressure was unchanged *in vivo*. We conclude that deficiency of cavin-1 affects the function of small arteries, but that opposing influences on arterial resistance balance each other such that systemic blood pressure in unstressed mice is well maintained.

## Introduction

Caveolae are invaginations in the plasma membrane that are important for cardiovascular structure, function and disease progression [1]. These membrane domains are enriched in cholesterol and serve as platforms for signaling events. Caveolin proteins are required for generation of caveolae [2], and both caveolin-1 and caveolin-3 can drive formation of these structures [3]. Recent work has identified cavin-1 (also known as polymerase and transcript release factor, PTRF) as a novel protein that is required in addition to the caveolins for caveolae to form [4], [5].

Caveolae deficient mice have been generated previously by ablation of either caveolin-1 or caveolin-3. Caveolin-1 knockout (KO) mice lack caveolae in all tissues except in striated muscle and exhibit metabolic, cardiovascular and pulmonary phenotypes [6]–[8]. The vascular phenotype comprises an increase in endothelial nitric oxide synthase activity [7]–[9], reduced myogenic tone [10], [11] and elevated α_1_-adrenergic contractility [11], [12]. This is associated with an unchanged, or slightly increased, systemic blood pressure [11], [13] and pulmonary arterial hypertension [9]. Functional studies on mice lacking cavin-1 are as yet scarce, but show that the mice exhibit a metabolic phenotype [5], and develop pulmonary arterial hypertension [14] in association with mild changes of the cytoarchitecture in the lung [14], [15]. An increased protein expression of Arginase-1 (Arg1) was moreover detected in the lung [14]. Arg1 converts arginine to ornithine, and an increased Arg1 level may thus reduce arginine availability and indirectly impair nitric oxide synthase (NOS) activity. Such a mechanism was proposed to contribute to pulmonary arterial hypertension in cavin-1 deficient mice, but whether peripheral arteries are similarly affected has not been tested.

Recent investigations suggest that both caveolin-1 and cavin-1 may have caveolae-independent effects [16], [17]. Indeed, caveolin-1 KO mice breed normally while cavin-1 KO mice, at least in our hands, exhibit a high perinatal lethality, suggesting important differences between the strains. The overall aim of the current study was to investigate the effect of cavin-1 deficiency on systemic arteries. We therefore performed detailed functional and morphometric analyses of small arteries from cavin-1 KO and wild type mice. Our findings indicate changes in α_1_-adrenergic responsiveness and pressure-induced myogenic tone.

## Materials and Methods

### Ethics Statement

All experimental protocols were approved by the local (Malmö-Lund) ethics committee at Lund University (M260-11). All mice were housed in an animal care facility at Lund University on a 12∶12 light:dark cycle and had access to food and water *ad libitum*. All efforts were made to minimize suffering.

### Mice

Cavin^+/−^ mice were purchased from Jackson Laboratories and bred as described earlier [14], [18]. Adult mice (4–5 months) were used for experiments and comparisons were made between knockouts (KO, ^−/−^) and wild type (WT, ^+/+^) littermate controls.

### Cavin-1-reporter staining

The targeting vector used to generate cavin-1-knockout mice contains a lacZ/neo cassette [5]. This allows for analysis of β-gal expression under control of the cavin-1 promoter. Staining for cavin-1 reporter expression was performed as described earlier [14], [18]. Briefly, blood vessels were micro-dissected, fixed with 4% paraformaldehyde and then incubated in a solution containing 150 mM NaCl, 2 mM MgCl_2_, 5 mM potassium ferricyanide, 5 mM potassium ferrocyanide, 40 mM citric acid, 12 mM sodium phosphate (pH 6.0) and 1 mg/ml X-gal (5-bromo-4-chloro-3-indolyl- beta-D-galactopyranoside, Sigma, St. Louis, MO) at 37°C. Photographs were taken using an Olympus (SZ61) dissection microscope (non-graded 0.67–4.5 × optical zoom) fitted with a digital camera.

### Electron microscopy

Arteries were perfusion fixed in 2.5% glutaraldehyde in 150 mM cacodylate buffer (pH 7.4) for 24 h. The tissues were then transferred to cacodylate buffer and further post-fixed in 1% osmium tetroxide for 2 h, block-stained with uranyl acetate, dehydrated and embedded. Sections were cut and examined in an electron microscope (JEOL 1230, Jeol, Tokyo, Japan). Digital photos were analyzed using Image J (NIH, Bethesda, MD, USA).

### Western blotting

Blood vessels were micro dissected and frozen in liquid N_2_ and stored at −80°C. To prepare a tissue homogenate the vessels were pulverized using a Qiagen Tissuelyser and dissolved in SDS sample buffer (62.5 mM Tris-HCl pH 6.8, 2% SDS (W/V), 10% (V/V) glycerol, 5% (V/V) mercaptoethanol) containing phosphatase and protease inhibitors (Sigma, St. Louis, USA). Protein concentration was determined using Bio-Rad DC™ protein assay and 20 μg protein was loaded in each well of the gels (4–15% or any KD, TGX Criterion, Biorad, Hercules, CA, USA). Proteins were transferred to nitrocellulose membranes (0.2 μm) using the Turboblot system (Biorad), blocked in casein blocking buffer (Biorad), washed in TBS-T (20 mM Tris-HCl, 0.5 M NaCl, 0.05% (v/v) Tween 20, pH 7.5), and incubated with primary antibodies: cavin-1 (1∶500) and cavin-2 (1∶500) (ab48824, ab113876 Abcam, Cambridge, MA), and cavin-3 (1∶500) (16250-1-AP ProteinTech group, Chicago, IL, USA), caveolin-1 (1∶10 000), -2 (1∶1000) and -3 (1∶10 000) (610407, 610684, and 610421, BD Transduction laboratories, San Jose, CA, USA), Arg1 (1∶1000) (GTX109242, Genetex, Irvine, CA, USA), connexin-40 (1∶1000) (ab1726, Millipore, Temecula, CA, USA) and -43(1∶1000) (ab11370, abcam), pS1177-eNOS (1∶500) (612392, BD Transduction Laboratories), smooth muscle α-actin (1∶1000) (A2547, Sigma, St. Louis, USA), calponin (ab46794, Abcam), SM-22 (1∶1000) (ab14106, Abcam)). The secondary antibodies used were HRP-linked anti-rabbit-IgG (1∶10 000) and anti-mouse IgG (1∶10 000) (Cell Signaling Technology, Danvers, MA, USA). Bands were detected using horseradish peroxidase-conjugated secondary antibodies and West Femto substrate (Pierce, Rockford, IL). An Odessey Fc instrument (LI-COR Biosciences) was used to detect chemiluminescence. All bands were normalized to a reference gene (GAPDH MAB374 (1∶1000), Millipore or HSP90 (1∶1000) 610418, BD Transduction Laboratories).

### Pressure myography

Small mesenteric arterial trees were freed from adherent fat in Ca^2+^-free HEPES-buffered Krebs (in mM: NaCl 135.5, KCl 5.9, MgCl_2_ 1.2, glucose 11.6, HEPES 11.6, pH 7.4) under a dissection microscope. The largest primary branch of each tree was cut and fitted on borosilicate glass capillaries (pulled using a L/M-3P-A (List Medical) to create a tip of ∼100 μm) in a pressure myograph chamber (Living Systems Instrumentation, Burlington, VT). Arteries were tightly secured with 6-0 silk sutures. An inverted microscope (Nikon) equipped with a CCD camera was used to visualize the vessel and VediView 1.2 software (Danish MyoTechnology) was used to monitor lumen and vessel diameter. The vessels were superfused with HEPES-buffered Krebs solution containing 2.5 mM CaCl_2_ and temperature was maintained at 37°C using a temperature controller (Living Systems). The bath solution was changed every 20 minutes. The pressure inside the artery was monitored by two pressure transducers mounted at the inflow and outflow lines. Intraluminal pressure was applied by a pressure servo controller (Living Systems). The vessels were allowed to equilibrate at 45 mmHg and 37°C for 1 h before experimentation. Arteries that showed signs of leakage were discarded. Before the start of each experiment the vessel was contracted using 60 mM KCl. To examine myogenic tone, intraluminal pressure was raised from 20 to 120 mmHg in 25 mmHg increments. The pressure was maintained for 7 min at each step and vessel diameter was recorded at the end of the 7 min period. This procedure was repeated with the same vessel after 30 min of incubation with 300 μM Nω-nitro-L-arginine methyl ester (L-NAME). To examine endothelium-mediated dilation the vessel was activated with phenylepherine (2 μM) and then dilated using acetylcholine (ACh: 1×10-9 → 1×10-4 mol/L). At the end of each experiment, the vessel was relaxed using Ca2+-free buffer supplemented with 2 mM EGTA. Myogenic tone (%) was expressed as [(D1 − D2)/D1] ×100, where D1 is the passive diameter in Ca^2+^-free buffer and D2 is the active diameter. Cross-sectional area was calculated as: π [(ID/2) + WT]2 − π (ID/2)2 where ID is inner diameter and WT is wall thickness in Ca2+-free solution at each pressure. Distensibility was estimated under relaxed conditions by determining changes in ID as a function of pressure normalized to the diameter at 10 mmHg.

### Wire myography

∼2 mm segments from the mesenteric artery branches remaining after dissection for pressure myography were mounted in myographs (610 M, Danish Myo Technology, Aarhus, Denmark) using stainless steel wire. No attempt to remove the endothelium was made. A basal tension of 5 mN was applied in a HEPES-buffered Krebs solution containing 2.5 mM CaCl_2_. Following equilibration, the preparations were contracted with 60 mM KCl for 7 min followed by relaxation for 25 min. The α_1_-adrenergic agonist cirazoline was added in a cumulative fashion (3 nM → 1000 nM) and each concentration was maintained for 7 min. Data was analyzed by integrating force over the stimulation period. The concentration-response curve was then repeated in an identical fashion after pre-incubation for 10 min with L-NAME (300 μM), ryanodine (10 μM), 18α-glycyrrhetinic acid (100 μM), a combination of charybdotoxin (100 nM) and apamin (300 nM), or ODQ (20 μM, 1*H*-[1], [2], [4]oxadiazolo[4, 3-a]quinoxalin-1-one). The lengths of the arteries were determined on completion of the experiments using a dissection microscope with an ocular scale and were used for normalization purposes.

### Hemodynamic measurements

Mice were anesthetized with 4% isoflurane (Isoflurane Forene; Abbot Scandinavia, Sweden) in room air in a small container and then transferred to a heating pad controlled by a rectal probe to keep temperature at 37±1°C (Temperature Control Unit HB 101/2; Panlab, Spain). After tracheotomy, anesthesia was maintained at 2% isoflurane by using a mouse ventilator (28025; Ugo Basile, Italy, tidal volume: 0.35 ml, frequency: 98/min). The left carotid artery was cannulated after the tracheotomy and pressure and heart rate was monitored on a polygraph. The right jugular vein was cannulated for infusion of L-NAME. Mean arterial blood pressure was recorded for at least 45 min before and after the infusion of L-NAME (a bolus of 5 μmol followed by infusion of 0.16 μmol/min).

### Statistics

Data are presented as means±SEM. For the *in vitro* vasodilatory and contractile dose responses, group differences were determined by repeated measures two way-ANOVA. Comparisons between the WT and KO groups were made using Student's t-test. Chi-square test was used for determining statistical significance between the frequencies of vasomotion in the different groups. P< 0.05 was considered significant.

## Results

### Distribution of Cavin-1 in the vascular system

**Figure 1:**
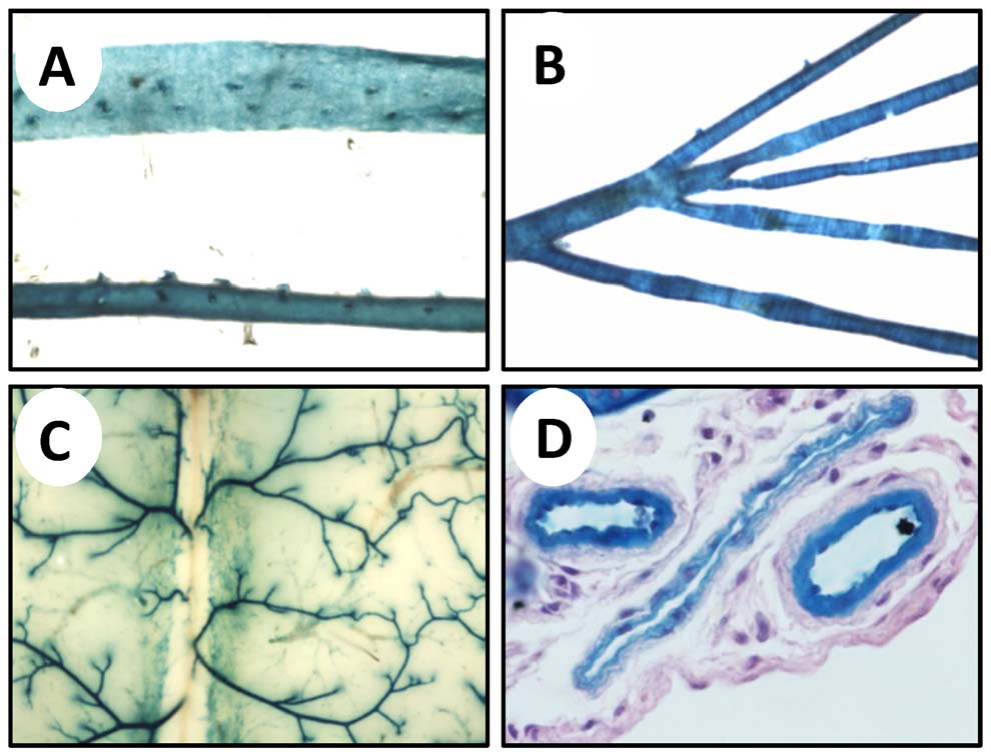
Cavin-1-reporter staining of systemic blood vessels. A) The aorta was dissected, fixed briefly in paraformaldehyde, and stained using x-gal. Bottom shows an intact aorta and top shows an opened aorta from the luminal side. B) Small mesenteric arteries with the superior mesenteric artery on the left. C) The vascular supply of the cerebral hemispheres. D) X-gal stained arteries and a vein in the prostate was paraffin embedded, sectioned, and counterstained using hematoxylin-eosin.

First we examined the distribution of cavin-1 in blood vessels using reporter staining. Reporter staining was high in arteries (Figure 1). The aorta (Figure 1A), a large conduit artery, and small mesenteric arteries (Figure 1B) were heavily stained. Incubation of whole brains with β-galactosidase substrate resulted in strong staining of cerebral arteries against a largely negative background (Figure 1C). Reporter staining was also evident in cryo-sections counterstained with haematoxilin-eosin, confirming a high expression of cavin-1 in the arterial endothelium and in smooth muscle, with less intense staining of the media in collapsed veins (Figure 1D shows cross-sections of two arteries and one vein in the prostate gland). Fainter staining of veins was also seen in the brain (Figure 1C) and in several other tissues.

### Cavin-1-deficient arteries have reduced levels of caveolae-associated proteins and lack caveolae

**Figure 2:**
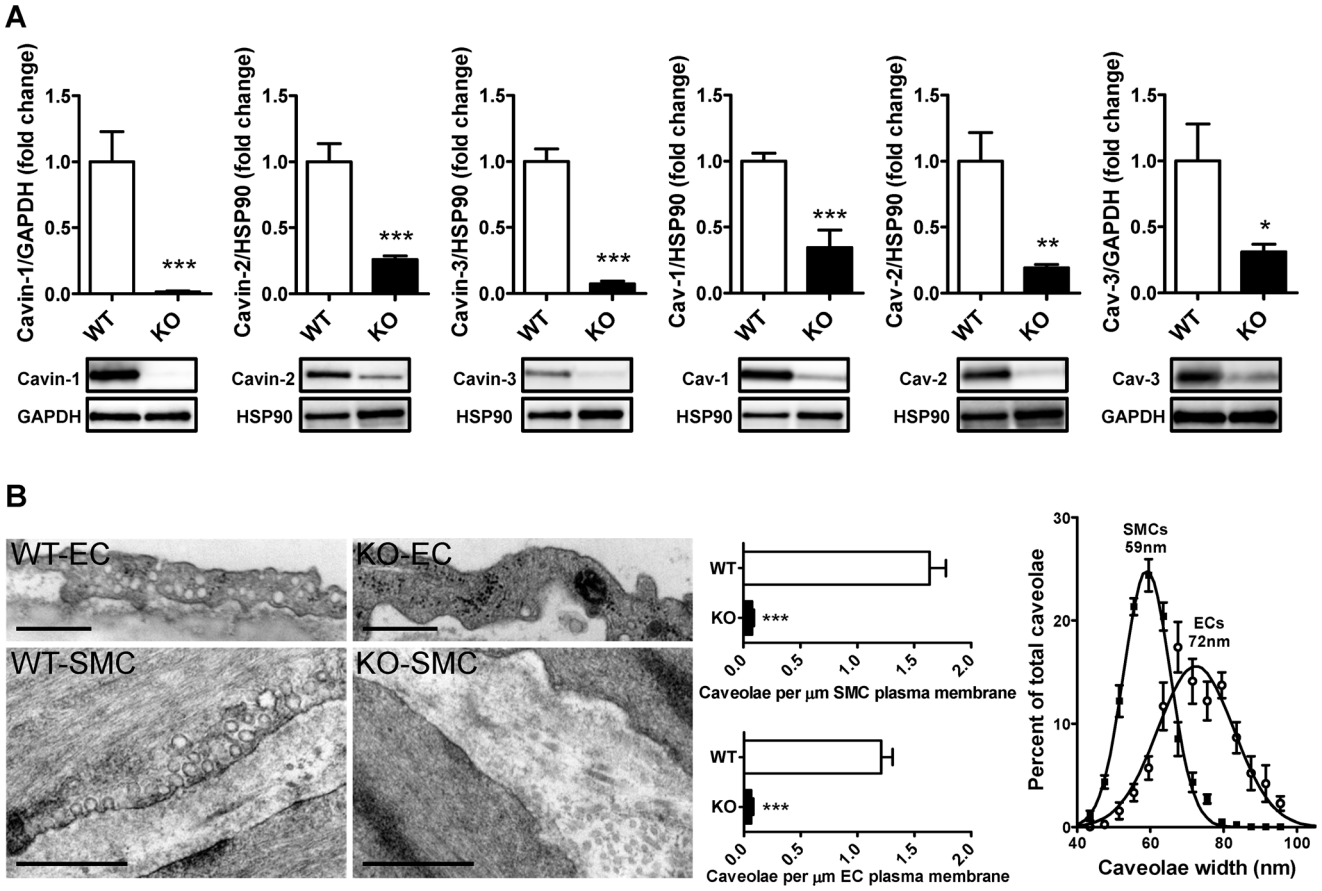
Reduced expression of caveolae-associated proteins in cavin-1^−/−^ mice. A) 20 μg of protein from small mesenteric artery lysates was subjected to western blotting for cavins and caveolins. GAPDH or HSP90 were used as loading controls. B) Electron micrographs showing membrane sections from wild type (WT) and cavin-1-deficient (KO) small mesenteric arteries. Top panels show endothelial cells (EC) and bottom panels show smooth muscle cells (SMC). Middle panel in B shows quantification of caveolae per μm membrane in endothelial cells (EC) and in smooth muscle cells (SMC) from WT and KO animals, respectively. Right panel shows size distribution of caveolae in endothelium compared to smooth muscle in WT arteries.

Caveolins and cavins were next measured in small mesenteric arteries using western blotting. Cavin-1 was absent as expected (Figure 2A). Furthermore, deletion of cavin-1 resulted in reduced expression of cavin-2 and -3 as well as of caveolin-1, -2 and -3 (Figure 2A). Similar results were found in the aorta except for cavin-2, which was unchanged (data not shown).

Transmission electron microscopy showed that lack of cavin-1 was associated with lack of caveolae in endothelial and smooth muscle cells of small mesenteric arteries (Figure 2B). Quantitative morphometry of micrographs (60 k magnification) from four wild-type (WT) and four cavin-1 KO mice confirmed this (Figure 2B, middle panel). Analysis of the diameters of caveolae in WT mice revealed that smooth muscle caveolae was somewhat smaller than endothelial caveolae (59 nm vs. 72 nm, Figure 2B, rightmost panel).

### Cavin-1 KO arteries have higher arginase-1 expression

**Figure 3:**
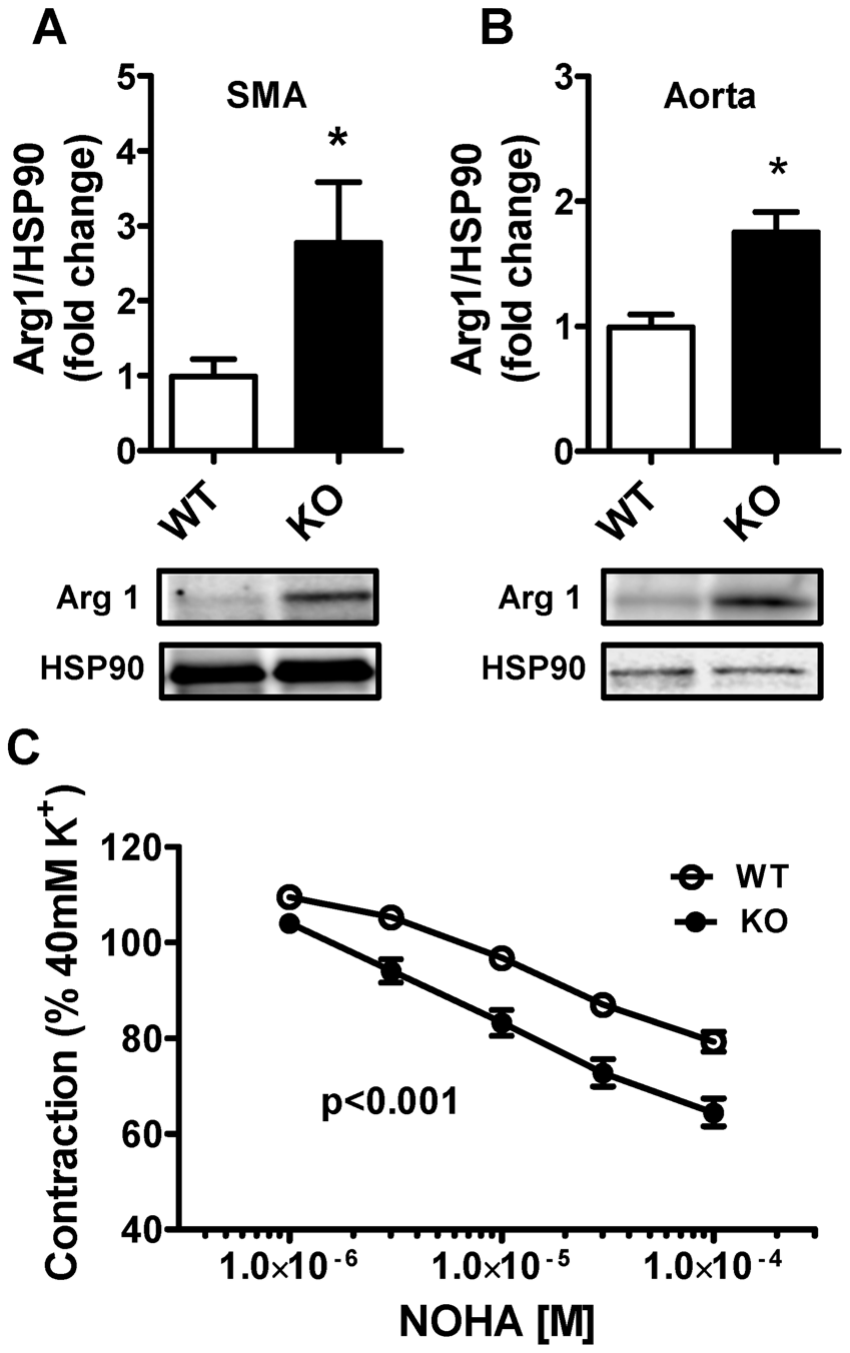
Elevated arginase1 expression and increased arginase inhibitor-induced relaxation of Cavin-1^−/−^ arteries. A and B) Western blotting for arginase 1 in small mesenteric arteries (SMA) A) and in aorta B) from wild type (WT) and cavin-1 knock out (KO) mice. C) SMA were mounted in a wire-myograph and pre-contraction with 40 mM KCl. Relaxation to increasing concentrations of NOHA, an arginase inhibitor, was then assessed.

We recently reported that the enzyme arginase-1 (Arg1) is elevated in the lungs of cavin-1 deficient mice [14]. Arg-1 limits nitric oxide (NO) production by conversion of arginine, the substrate for eNOS, to ornithine. To address if Arg1 is elevated in systemic arteries we measured protein expression in the aorta and in mesenteric arteries and found a 2-3 fold elevation in cavin-1 KO compared to WT mice (Figure 3A and B). Small mesenteric arteries with intact endothelium were next mounted in wire myographs and relaxation by the arginase inhibitor NOHA was examined. To minimize the influence of endothelium-dependent hyperpolarization, arteries were stimulated with 40 mM K^+^ followed by cumulative addition of NOHA. NOHA evoked larger relaxation in KO compared to WT mice (Figure 3C) suggesting increased Arg-1 activity and/or elevated basal NO production in cavin-1 KO mice.

### Increased α_1_-adrenergic contractility in small mesenteric arteries from cavin-1-deficient mice

**Figure 4:**
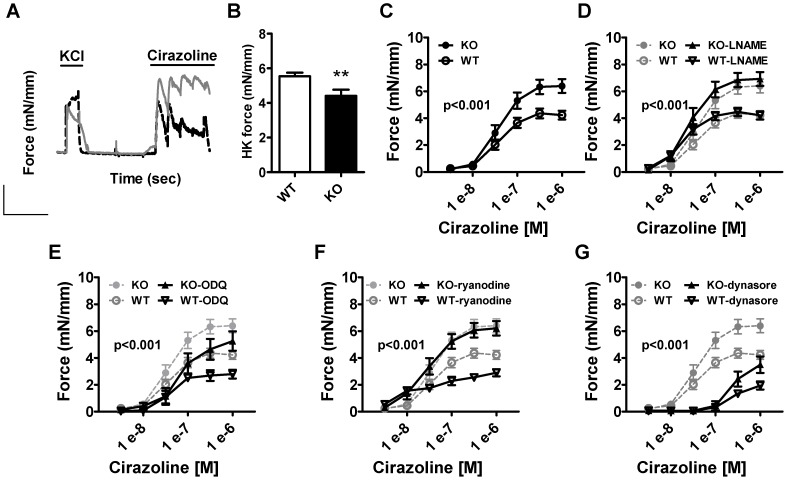
Increased α_1_-adrenergic contraction in small mesenteric arteries from cavin-1-deficient mice. A) Original traces showing contraction to 60 mM KCl and to cumulative addition of cirazoline in one representative wild type (WT; black) and knock out (KO; grey) mouse. Concentration-response curves for the α_1_-adrenergic agonist cirazoline (C, control conditions; D, +300 μM of the NOS-inhibitor L-NAME; E, in the presence of 20 μM ODQ; F, in the presence of 10 μM ryanodine; G, in the presence of 20 μM dynasore) in small mesenteric arteries shows increased α_1_-adrenergic contractility in the absence of cavin-1. As a reference, contraction to cirazoline in WT and KO is replicated in grey in panel D, E, F and G. The statistical difference between WT-drug and KO-drug is indicated by the p-value in the graphs. Panel B shows integrated force in response to depolarization (60 mM KCl).

To further assess contractility in the absence of cavin-1 we stimulated small mesenteric arteries with increasing concentrations of the α_1_-adrenergic agonist cirazoline and measured absolute force per length of the arterial segment. Contraction was increased at the highest cirazoline concentrations (Figure 4A and C). In contrast, contraction in response to 60 mM KCl, which represents maximal activation, was reduced (Figure 4B). To rule out an impact of nitric oxide synthase (NOS) activity during agonist stimulation, we did the same experiment in the presence of the NOS-inhibitor L-NAME and the guanylyl cyclase inhibitor ODQ. The difference in α_1_-adrenergic contraction between genotypes was well maintained in these conditions (Figure 4D, E).

Since molecules involved in calcium handling are associated with caveolae we examined if the difference in contractility between WT and KO mice was dependent on Ca^2+^-sparks. When Ca^2+^-sparks were inhibited by ryanodine we still found a significantly elevated α_1_-adrenergic contractility in cavin-1 KO compared to WT arteries (Figure 4F). We finally tested if dynasore, a dynamin inhibitor that interferes with trafficking of caveolae, would eliminate the increased adrenergic response in cavin-1 KO arteries. Dynasore concentration-dependently inhibited force irrespective of mode of stimulation (not shown) and when the concentration was titrated to allow for some force development, a significant difference in cirazoline-induced contraction was still observed (Figure 4G). This suggested that loss of caveolar trafficking was not responsible for the increased adrenergic response.

### Impaired myoendothelial connectivity does not underlie increased α_1_-adrenergic contractility

**Figure 5:**
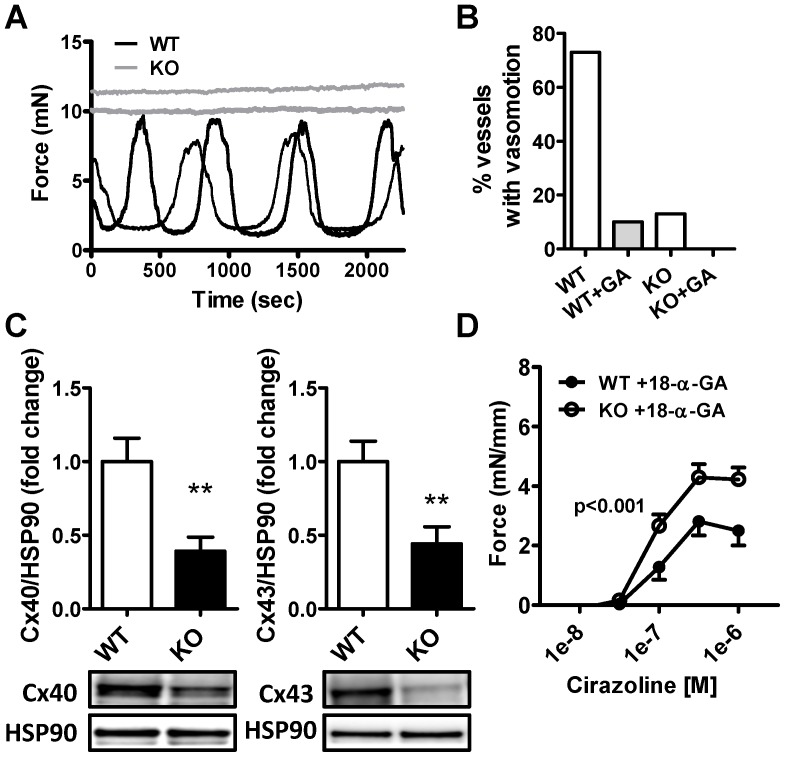
Cavin-1 deficient mice exhibit reduced vasomotion. Panel A) shows original traces from two wild type (WT; black) and two knock out (KO; grey) arteries, mounted in a wire-myograph, after prolonged (1 h) incubation with cirazoline (0.1 μM). Note the absence of rhythmic force oscillations (vasomotion) in KO. Panel B) shows the fraction of vessels exhibiting vasomotion in WT and KO in the presence and absence of the gap junction blocker 18-α-glycyrrhetinic acid (18-α-GA). Panel C) shows expression of connexin 40 and 43 in the aorta and D) shows that 18-α-GA does not reduce the difference between WT and KO in α_1_-adrenergic contraction.

Vasomotion represents phasic fluctuations in arterial tone elicited by prolonged agonist stimulation [19], [20]. We noted in the above experiments that vasomotion occurred more seldom in cavin-1-KO arteries (Figure 5A shows examples of two WT and two KO arteries run in parallel). We therefore quantified the presence of vasomotion and found that it was significantly reduced in KO arteries (10% vs. 73%, p<0.001, Figure 5B). Vasomotion depends on myoendothelial connectivity via connexins [21], and we found that expression of connexins −40 and −43 was decreased in aortic lysates from cavin-1 KO mice (Figure 5C). 18-α-glycyrrhetinic acid (18-α-GA), an inhibitor of myoendothelial connectivity, moreover reduced the fraction of arteries that exhibited vasomotion in WT (from 73% to 13%, p<0.001, Figure 5B). 18-α-GA similarly reduced the proportion of KO vessels with vasomotion (from 10% to 0%, p<0.05), but the significant difference between WT and KO was eliminated (p = 0.13). We therefore proceeded to test if the difference in integrated α_1_-adrenergic contractility between WT and KO might be due impaired myoendothelial connectivity and reduced endothelial derived hyperpolarization (EDH). However, neither 18-α-GA (100 μM) nor charybdotoxin/apamin, a drug combination that blocks Ca^2+^ activated potassium channels and interferes with EDH, eliminated the difference in α_1_-adrenergic contraction (Figure 5D and data not shown).

### Altered geometry of small cavin-1-deficient arteries

**Figure 6:**
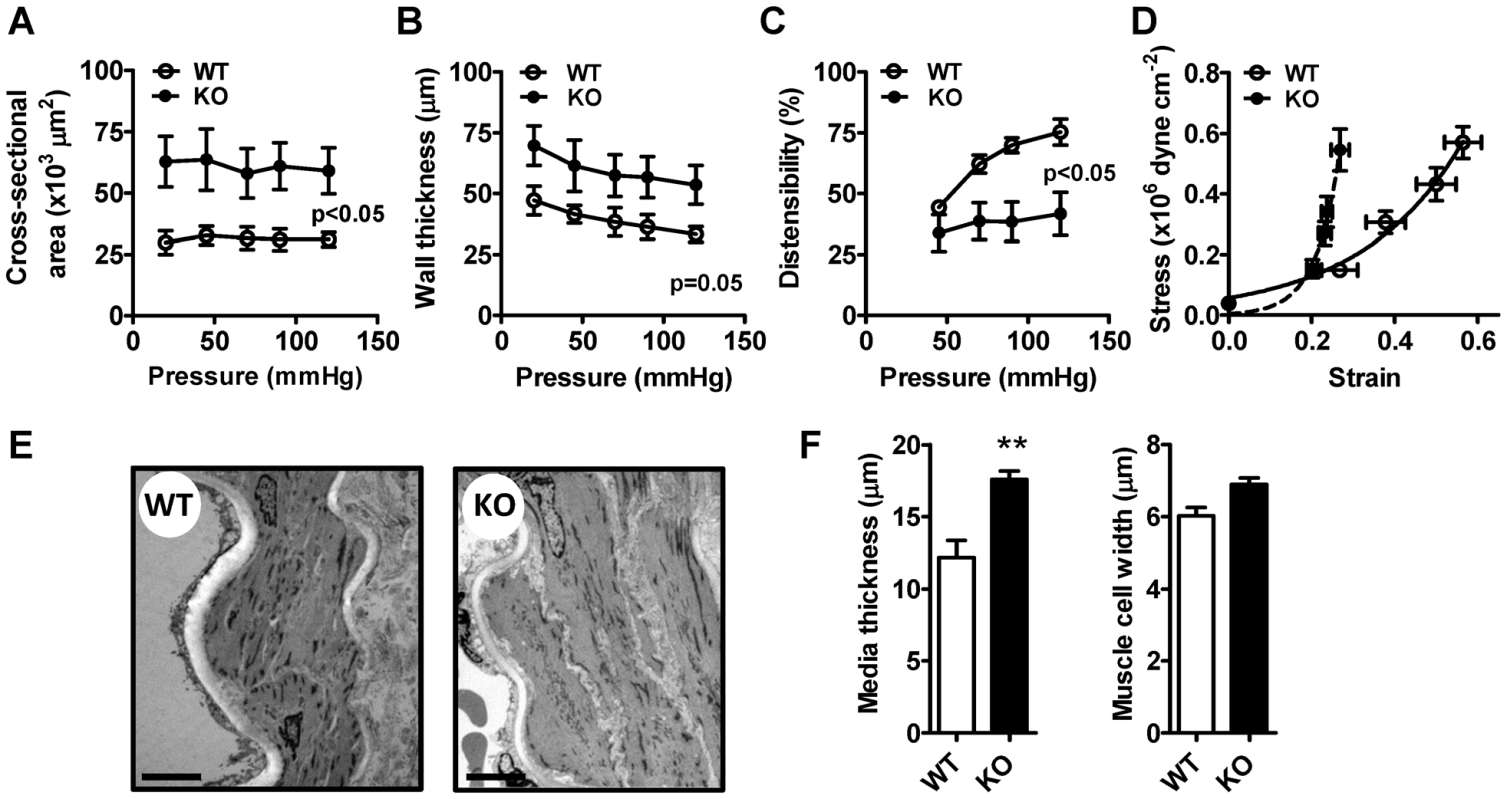
Arterial remodeling in cavin-1 deficient mice. Cross-sectional area (A), wall thickness (B), arterial distensibility (C) and stress-strain relationship (D) were assessed in a pressure myograph under Ca^2+^-free conditions. Measurements of vascular wall dimensions by electron microscopy were done using perfusion fixed vessels. Representative electron micrographs are shown in (E) and measurements of media thickness and muscle cell width in arteries from six wild type (WT) and six knock out (KO) animals is shown in (F).

To investigate if altered smooth muscle phenotype could explain the increased contractility in cavin-1 KO arteries we assessed the levels of contractile markers by western blotting. We found that smooth muscle myosin heavy chain, smooth muscle α-actin, calponin and SM22, were unchanged relative to GAPDH (data not shown). We therefore considered the possibility that media thickness was increased in KO versus WT arteries. To ascertain that our measurements were made at identical distending pressures we used maximally dilated and live vessels mounted in a pressure myograph. An increased cross-sectional area of the vessel wall was observed at all pressures in cavin-1 KO compared to WT arteries (Figure 6A). Arterial wall thickness was moreover increased at physiological pressures (Figure 6B, p = 0.05 using repeated measure ANOVA, p<0.05 using Student's t-test at 120 mm Hg). These changes were associated with a reduced arterial distensibility (Figure 6C), but wall to lumen ratio was unchanged (not shown). A steeper stress-strain relationship was also found (Figure 6D). Because the distinction between media and adventitia is ambiguous in the frames captured by the pressure myograph camera we also determined the media thickness using transmission electron microscopy. The media thickness, defined as the distance between the internal and external elastic laminae, was increased in KO arteries (Figure 6E and F). No statistically significant differences in inner lamina thickness, outer lamina thickness or muscle cell width were observed (Figure 6E and data not shown).

### Reduced myogenic tone in cavin-1-deficient arteries

**Figure 7:**
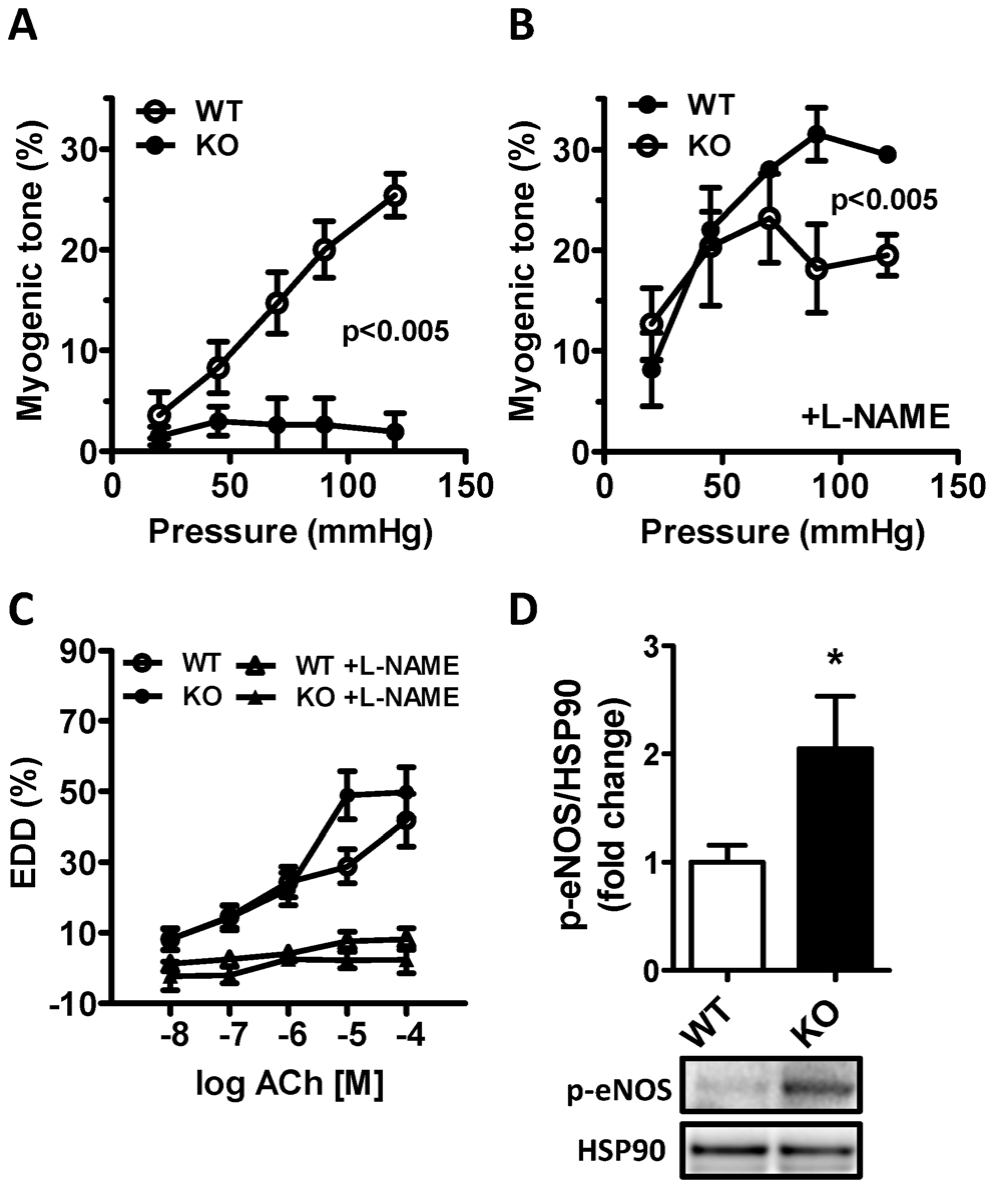
Reduced myogenic tone in cavin-1^−/−^ mice. Small mesenteric arteries were mounted in pressure myographs and their diameters at different pressures were recorded. A) Shows myogenic tone, calculated as described in Material and Methods, in control conditions. B) Shows myogenic tone in the presence of L-NAME (100 μM). Endothelium-dependent dilatation with acetylcholine is shown in (C) and phosphorylation of e-NOS is shown by western blotting in (D) in wild type (WT) and knock-out (KO) mice.

Prior work indicated a reduction of myogenic tone in caveolin-1-deficient arteries. We therefore measured myogenic tone in the absence of cavin-1 using pressure myography. We found that myogenic tone was reduced in the absence of cavin-1 (Figure 7A). A part of the difference appeared to depend on nitric oxide synthase activity because the difference at lower pressures (<95 mmHg) was eliminated by L-NAME (compare Figure 7A and B). We also examined acetylcholine-induced (endothelial-dependent) dilatation but there was no difference between WT and cavin-1 KO (Figure 7C). However, we found an increase of phosphorylated (activated) eNOS in KO arteries (Figure 7D).

### Blood pressure is normal in cavin-1 deficient mice

**Figure 8:**
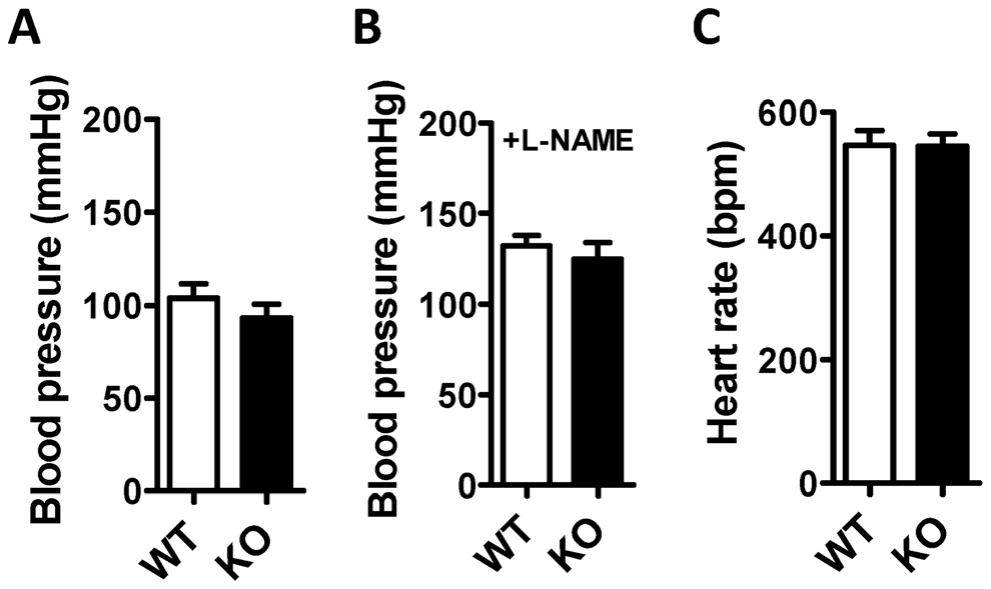
Unchanged mean arterial blood pressure in cavin-1-deficient mice. Mean arterial blood pressure was measured in the carotid artery of anaesthetized mice in control conditions (A) and after infusion of the nitric oxide synthase inhibitor L-NAME (B). Panel C shows heart rate in control conditions.

Our work so far demonstrates changes in arterial function in the absence of cavin-1, some of which would favor hypertension and some of which would favor hypotension. We therefore directly measured systemic blood pressure and found no difference between WT and cavin-1 KO, neither in the basal state nor after infusion of L-NAME, (Figure 8A–B). Heart rate was moreover similar (Figure 8C).

## Discussion

Cavin-1 is highly expressed in the vasculature and is present both in the endothelium and in the smooth muscle layer [5], [14], [22]. Cavin-1 is a cytosolic protein that interacts with caveolin-1 at the plasma membrane and aids in formation of caveolae, structures that play an important functional role in the vascular wall. However, cavin-1 may also play roles that are independent of caveolae. Indeed, the protein was first identified as a nuclear polymerase and transcript release factor [23] and in prostate cancer cells cavin-1 was found to act independently of caveolae [16], [17]. In view of these diverging influences it was important to investigate the effect of cavin-1 deletion on arterial function. To the best of our knowledge our study provides the first functional characterization of cavin-1-deficient arteries and we demonstrate wide-reaching changes in both structure and function.

In agreement with earlier reports focusing on other tissues, disruption of the cavin-1 gene resulted in loss of caveolae and in decreased levels of caveolae-associated proteins [5], [14], [18], [24]. We found this to be true in both small mesenteric arteries (SMA) and in the aorta. A notable exception was cavin-2, which was reduced in small mesenteric arteries but not in the aorta. Previous studies have shown that cavin-2 is necessary for generating caveolae in some tissues but not in others [22]. Because disassembly of the caveolar scaffold is known to destabilize its components, our findings suggest that cavin-2 may be an important component of the scaffold in small arteries but not in the aorta. This is of interest given differences between the aorta and small arteries, both in regard to function but also regarding susceptibility to disease. A detailed analysis of caveolae diameters in smooth muscle and endothelial cells from WT mice revealed a different size distribution of caveolae in these cell types. Whether this is due to differential expression of caveolae-associated remains to be elucidated.

In a recent study we found that mice lacking cavin-1 develop pulmonary arterial hypertension associated with elevated arginase1 (Arg1) expression in the lung [14]. Here we tested if Arg1 expression was altered in peripheral vessels of cavin-1 KO mice. Indeed, we found the arginase1 protein level to be higher in cavin-1 KO mice and that NOHA, an arginase inhibitor, induced a greater vascular relaxation, suggesting elevated basal NO production. Increased Arg1 activity is expected to enhance vascular contractility by depleting the substrate for NO production. We therefore examined the contractile response to α-adrenergic stimulation and found it to be increased. This is similar to what has been observed in caveolin-1 KO mice [11], [12], [25], indicating considerable phenotypic overlap in this regard. As expected, pharmacological experiments using L-NAME (a NOS inhibitor) and ODQ (a protein kinase G inhibitor) did not support the acute involvement of the nitric oxide pathway in the differential response to α-adrenergic activation in the wire-myograph setup. Similar result were obtained after addition of ryanodine, a drug inhibiting Ca^2+^ sparks which are elementary Ca^2+^ release events that hyperpolarize the membrane by activating Ca^2+^-activated potassium channels [26]. We could further show that inhibition of caveolar trafficking using dynasore did not abolish the difference between WT and cavin-1 KO. Taken together, these experiments show that deletion of cavin-1 results in an increased α_1_-adrenergic contractile response which is independent of acute production of NO/cGMP, of ryanodine receptor function and of caveolar trafficking.

A striking alteration in cavin-1 KO arteries was the lack of spontaneous rhythmic oscillations in force on prolonged stimulation with cirazoline. This phenomenon, referred to as vasomotion, is considered to depend on myoendothelial connectivity via gap junctions [19]. Work on caveolin-1-deficient arteries has demonstrated reduced oscillatory contractions, impaired myoendothelial connectivity and reduced expression of connexins 37, 40 and 43 [27]. Here we observed reductions of connexin 40 and 43 in the absence of cavin-1. Inhibition of myoendothelial signaling by 18-α-glycyrrhetinic acid (18-α-GA) reduced vasomotion in WT arteries to the level seen in KO arteries. This argues strongly that impaired myoendothelial connectivity underlies the observed difference in vasomotion. Because myoendothelial connectivity plays an essential role for endothelium-dependent hyperpolarization (EDH) we reasoned that an impaired EDH might underlie increased α_1_-adrenergic contractility. However, 18-α-GA did not eliminate the difference in integrated force during α_1_-adrenergic stimulation.

Because the increased α-adrenergic contraction resisted multiple pharmacological interventions we examined if it was due to an increased muscle mass. Using maximally dilated vessels *ex vivo* we were able to demonstrate increased cross-sectional area and arterial wall thickness and this was further confirmed using electron microscopy. It therefore seems reasonable to conclude that increased α_1_-adrenergic contraction in cavin-1-deficient vessels scales with an increased media thickness, but additional mechanisms cannot be ruled out. For instance, Ca^2+^ sensitization mechanisms [28] may be different, and in a previous study on caveolin-1-deficient arteries we detected increased Rho-activation and protein kinas C-induced contraction [12]. It should be noted that depolarization-induced contraction was impaired and that this difference would be greater if one were to correct for the increased media thickness. We can only speculate on the basis of this reduction, but it is of interest that microarray data [14] indicated a reduction of Rem1 in lungs from cavin-1-deficient mice. Rem1 is a monomeric GTPase that controls L-type Ca^2+^ channel density [29] and Ca^2+^ transient amplitudes in the heart [30]. Rem1 may possibly play a similar role in the arterial wall. An effect on Ca^2+^ channel gating would preferentially affect depolarization-induced contraction because cirazoline responses are comparatively resistant to L-type channel inhibition [31].

In cavin-1 KO mice we found an almost complete lack of myogenic tone, which was partly restored in the presence of L-NAME. This mimics the situation in certain arteries where pressure-stimulated constriction can be unmasked by nitric oxide synthase inhibition [32]. It is also in keeping with earlier observations in caveolin-1 deficient mice [10], [11]. Our findings suggest that an elevated local NO production inhibits the myogenic response. In keeping with this possibility we also found increased eNOS phosphorylation. It is unclear why increased NO production was not apparent in the wire myograph setting, but it is well known that the different experimental setups deliver diverging results in many regards [33], including e.g. sensitivity to contractile agonists. Earlier work has established that caveolin-1 inhibits eNOS activity *in vitro* [1], [2], and several previous investigations have demonstrated that caveolin-1-deficient mice have increased production of nitric oxide [7]–[9]. It was therefore not unexpected that cavin-1 deficient would exhibit some signs of elevated NO production, rather, its impact was surprisingly modest. Prior studies have also demonstrated a role of Ca^2+^ sparks in the regulation of myogenic tone [32]. In view of the small effect of ryanodine in our wire myograph experiments we did not test this substance in the pressure myograph setting, but earlier work has documented a difference in spontaneous transient outward currents, the effector mechanism by which Ca^2+^ sparks cause relaxation [7], in the absence of caveolin-1. Here, on the other hand, we demonstrated a greater influence of arginase in caveolae-deficient blood vessels, and we believe that this effect, by competing with eNOS for L-arginine, effectively masks an underlying increase in eNOS activity in some of our experimental paradigms. Opposing influences from increased arginase and eNOS activity, respectively, may also explain the preserved endothelium dependent vasodilatation in cavin-1-deficient mice.

A difference in arterial geometry similar to that found here has been demonstrated earlier in caveolin-1 deficient mice[11], [12], [25], [34], arguing that the phenotypic overlap between caveolin-1-deficient and cavin-1-deficient arteries is considerable also in this regard. The mechanism underlying the altered structure in either model is not known, but it has been argued that chronic breakdown of myogenic tone elicits a stereotypic change in geometry that is an attempt to offset an elevated wall stress through an increased wall thickness[35]. Another possibility is that ablation of caveolae affects growth signaling pathways. This possibility is supported by studies showing increased ERK1/2 activation and proliferation in caveolin-1-deficient smooth muscle cells [36]. Both of these putative mechanisms are attractive from a theoretical point of view, but they are difficult to address rigorously in vivo. It should be noted that the change in structure in caveolae-deficient arteries may be age-dependent as Haussman et al. [34] found that aging of caveolin-1-KO mice resulted in a reduction of cross-sectional area, which was opposite to the effect of ageing in WT mice. This argues that the underlying mechanisms may be complex. That study also found an unchanged wall to lumen ratio similar to our current findings. It should finally be noted that the increased media thickness likely contributes to the reduced distensibilty and to the increased steepness of the stress-strain relation in cavin-1 KO arteries, but additional mechanisms such as an increased adventitial thickness or altered matrix properties cannot be ruled out.

Impaired myoendothelial connectivity, reduced myogenic tone and increased α_1_-adrenergic contraction are expected to influence arterial resistance, and hence blood pressure, in opposing directions. In keeping with this prediction we directly demonstrate that arterial blood pressure in the systemic circulation is unchanged in the absence of cavin-1. This contrasts with the situation in the pulmonary circulation where blood pressure is increased [14]. A potential concern with our in vivo experiments is that anesthesia reduces blood pressure. We chose the method to measure blood pressure to allow for direct comparisons with our prior data on caveolin-1-deficient mice, and it has previously been demonstrated that add-on treatment with isoflurane does not affect the comparison of blood-pressure between wild-type and caveolin-1 knockout mice (discussed by Rahman and Sward, 2009)[37]. Thus, while blood pressure might be somewhat higher in conscious mice there is no reason to believe that isoflurane anesthesia has a differential impact on blood pressure depending on genotype. All in all, our study demonstrates a critical role of cavin-1 in vascular structure and function, but the influences on arterial resistance cancel each other such that arterial blood pressure remains unaltered in homeostatic conditions.
